# Supplementary material for: High Prevalence of Clonal Reproduction and Low Genetic Diversity in Scutellaria floridana, a Federally Threatened Florida-Endemic Mint
Source: Plants (Basel). 2023 Feb 17;12(4):919. doi: 10.3390/plants12040919 (PMC9964081; doi:10.3390/plants12040919)
Supplement: Supplementary file 1 [file plants-12-00919-s001.zip › plants-2123369-supplementary.pdf]

SUPPLEMENTAL MATERIALS

Table S1: Percent change in NLCD land cover categories between 2001 and 2019 across *S. floridana*'s range, on managed lands within *S. floridana*'s range, within 500 m of populations confirmed to be extant, and within 500 m of populations in which we were unable to locate any individuals.

Land Cover Categories	Across Range	Managed Lands	Confirmed Extant	Failed to Find
Open Water	0.12	-0.04	0.01	-0.02
Developed, open space	-2.79	-0.01	0.06	-0.53
Developed, low intensity	3.07	0.01	0.04	0.05
Developed, medium intensity	20.61	0.03	0.10	0.51
Developed, high intensity	14.04	0.00	0.00	0.00
Barren Land	1.54	0.00	0.07	0.01
Deciduous Forest	-33.07	-0.03	0.01	-0.02
Evergreen Forest	-13.70	0.00	-1.23	-1.01
Mixed Forest	17.96	0.00	0.00	0.00
Shrub/Scrub	19.22	0.10	0.41	1.58
Grassland/Herbaceous	38.47	-0.09	0.60	-0.66
Pasture/Hay	-1.69	0.00	0.00	-0.02
Cultivated Crops	0.67	0.00	0.00	-0.02
Woody Wetlands	-2.28	1.75	-0.04	-3.20
Emerging Herbaceous Wetlands	21.85	-1.73	-0.04	3.33

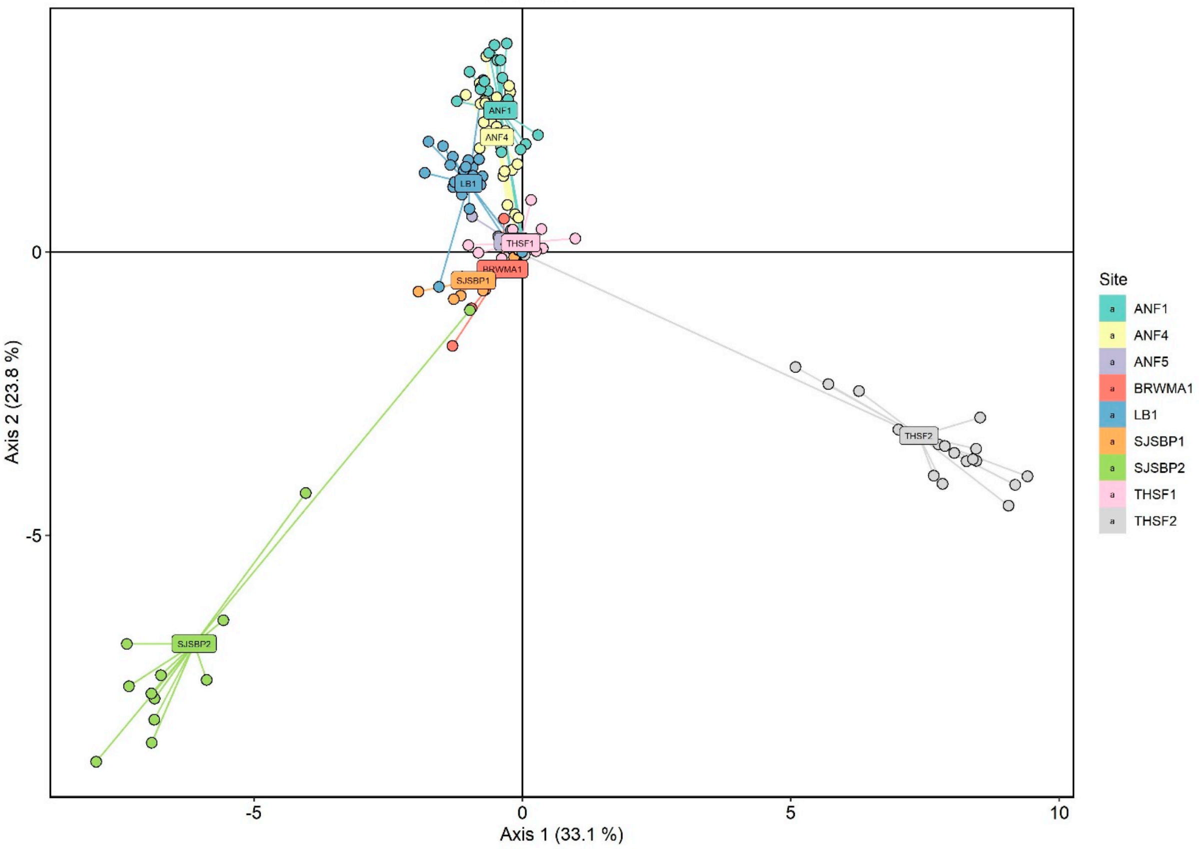

Figure S1. DAPC with excluded outlying populations (ANF2, ANF 3, and BRWMA2) using 20 principal components and eight axes. Circles represent individuals and labels are placed on the centroid (average) position for each population.

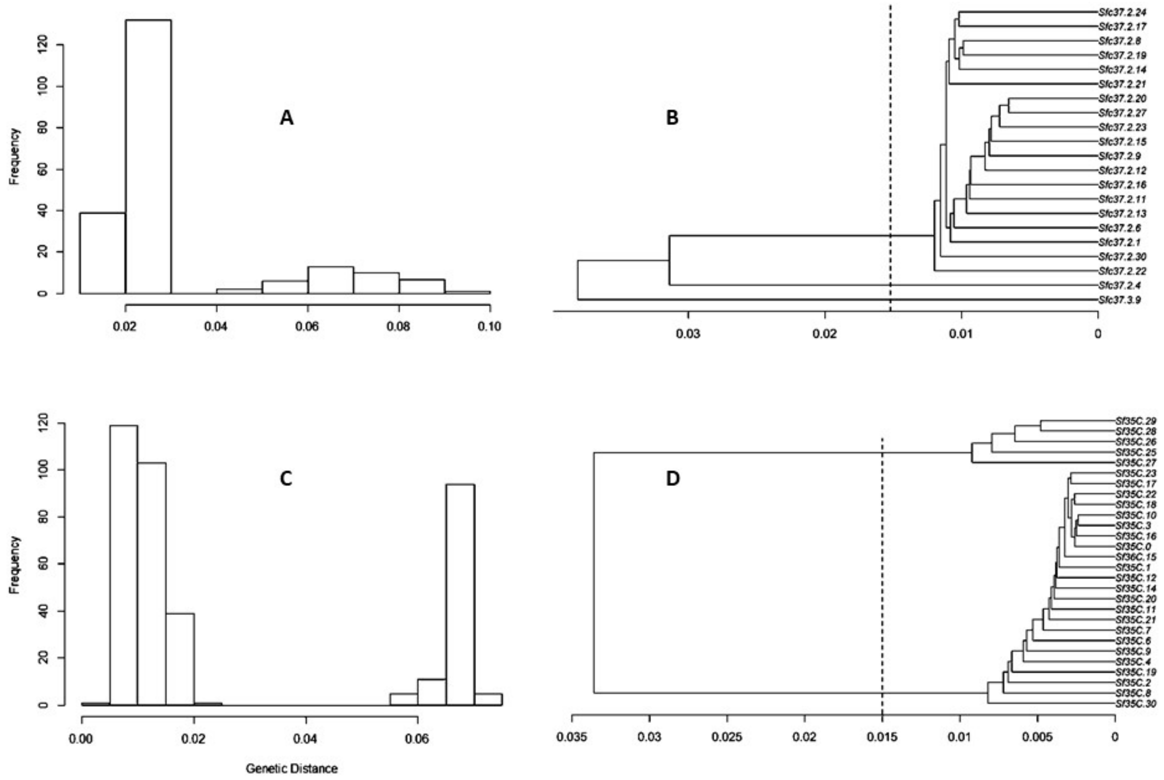

Figure S2. Histogram of genetic distances and UPGMA trees for 5 m circular plots established in ANF4 (A and B) and SJSBP2 (C and D). Distance threshold to distinguish individuals was set at 0.03 for both plots (A and C). Dotted line (B and D) represents distance threshold after which individuals are no longer genetically unique.
